# A Random Angular Bend Algorithm for Two- Dimensional Discrete Modeling of Granular Materials

**DOI:** 10.3390/ma12132169

**Published:** 2019-07-05

**Authors:** Zhenyu Wang, Lin Wang, Wengang Zhang

**Affiliations:** 1 School of Civil Engineering, Chongqing University, Chongqing 400045, China; 2 Key Laboratory of New Technology for Construction of Cities in Mountain Area, Chongqing University, Chongqing 400045, China; 3 National Joint Engineering Research Center of Geohazards Prevention in the Reservoir Areas, Chongqing University, Chongqing 400045, China

**Keywords:** particle shape, discrete element method, random angular bend, overlap detection

## Abstract

Generation of particles with irregular shape and the overlap detection are crucial for numerical simulation of granular materials. This paper presents a systematic approach to develop a two-dimensional random particle model for numerical simulation of granular materials. Firstly, a random angular bend (RAB) algorithm is proposed and coded in Python to simulate the geometric model of individual particle with irregular shape. Three representative parameters are used to quantitatively control the shape feature of generated polygons in terms of three major aspects, respectively. Then, the generated geometrical models are implemented into particle flow code PFC^2D^ to construct the clump library. The clumps are created via the mid-surface method. Besides, an overlap detection algorithm is developed to address the difficulties associated with spatial allocation of irregularly shaped particles. Finally, two application examples are adopted to validate the feasibility of the proposed algorithm in the numerical modeling of realistic granular materials. The study provides a solid foundation for the generation and simulation of the granular materials based on angular bend theory.

## 1. Introduction

Granular materials are conglomerations of discrete solids [1], which are widely used in various engineering fields, such as geotechnical engineering, mining engineering, and food and pharmaceutical industries. Due to the discontinuous nature and irregularity of particle shape, the study of properties of granular materials remains an open issue. In the study of granular material behavior, some experimental tests were conducted to analyze the effect of particle shape and size on the mechanical and physical properties of granular materials [2,3]. However, it is difficult to precisely control particle shape and size in these tests. Besides, conventional experimental tests cannot provide sufficient microscale information of samples, such as interparticle forces, particle velocities, etc.

Overcoming the disadvantage of model test in these respects, the numerical analysis method is commonly accepted by many researchers in the last decade. The discrete element method (DEM), originally developed by Cundall in 1971 [4,5], provides a convenient way to obtain particle information on multiple scales, and gradually becomes an effective tool to study the mechanical behaviors of granular materials [6,7,8,9]. To investigate the effect of particle shape, various shapes have been created to approximate the realistic shape of particles (such as circles and spheres [10,11,12,13], ellipses and ellipsoids [14,15], cylinders [16], polygons, and polyhedrons [17,18,19]). However, a considerable number of them are idealized geometric shape, which is unable to capture the behavior of actual or natural granular materials better.

To precisely generate arbitrary-shaped particle in two-dimensional simulation, many methods have been proposed in the literature. Typical approaches include R(θ) method [20], Voronoi grain-based method [21,22], digital image-based method [23], image-based clump library method [24,25], Fourier-based method [26,27,28], etc. However, the R(θ) method is only applicable for star-like particles [29,30]. The Voronoi grain-based method has limited capability in precisely controlling generated particle shape and size. During the modeling of granular materials based on digital image technology, it is difficult to obtain digital images of the whole research area or obtain the digital images of idealized section for specified research purpose. Image-based clump library method overcomes the disadvantage of above method and provides a new method to create virtual specimens possessing various shapes and particle gradations. However, tremendous efforts are required to develop the clump library (a clump is a rigid collection of n rigid spherical pebbles, which are unit-thickness disks in PFC^2D^). Among various Fourier-based approaches, the shape function R(θ) based method is widely used in shape analysis and generation. However, this method is only applicable for star-like particles. For nonstar-like particles, the radial line may have multiple intersections with the boundary of particle contour [30]. Compared with the methods above, the shape function φ(l) (Z-R shape function) based method which is developed by Zahn and Roskies (1972) has distinct advantages in precisely representing the realistic particles [31]. However, there is limited research on the random generation of irregular particles based on this theory.

**Figure 1:**
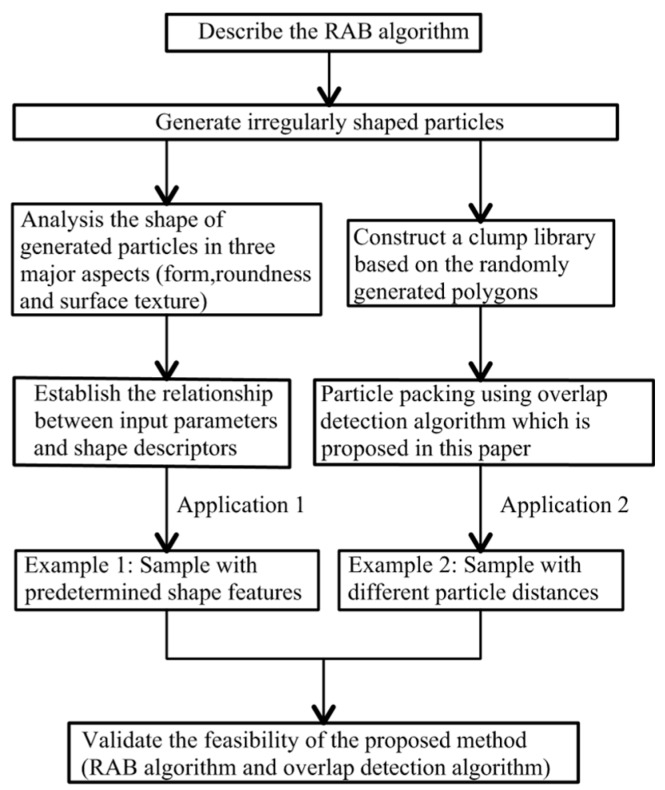
Flowchart of this paper.

Inspired by the theory of Z-R shape function, this paper presents a novel method to generate two-dimensional random particle model for the numerical simulation of granular materials. The flowchart of this paper is shown in Figure 1. Firstly, a random angular bend (RAB) algorithm is proposed and coded in Python [32] to simulate the geometric model of individual particle with irregular shape. Three representative parameters are used to quantitatively control the shape feature of generated polygons in term of three major aspects, namely, the form, roundness, and surface textures, respectively. The geometric models are imported into PFC^2D^ [33] to generate realistic granular media. Clumps consisting of pebbles are created based on the mid-surface method [34] and used to construct clump library. An overlap detection algorithm is developed to address difficulties associated with spatial allocation of irregularly shaped particles. Finally, two application examples are further employed to show the feasibility of proposed algorithm for the numerical modeling of realistic granular materials.

## 2. Particle Generation

### 2.1. Z-R Shape Function

**Figure 2:**
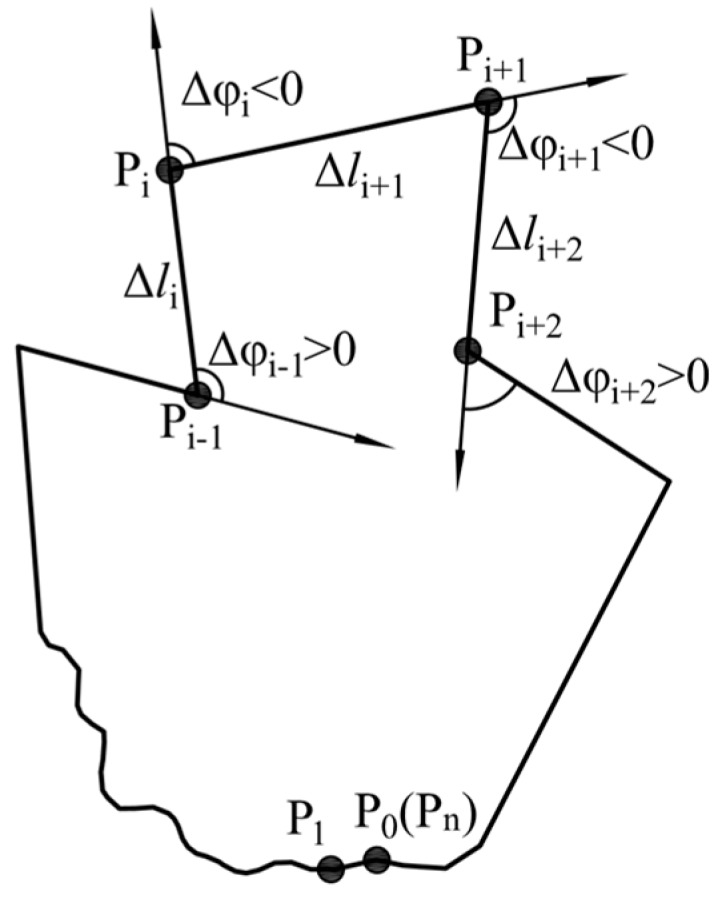
Description of Particle shape using the result of the Z-R shape function.

Shape function φ(*l*) was introduced by Zahn and Roskies (1972) to represent the shape of closed polygonal curve in term of arc length *l* and cumulative angular bends *φ*. To describe the shape of a polygon, which is made up of n vertices P_0_, P_1_,…, P_n_ with angular bends Δ*φ*_0_, Δ*φ*_1_,…, Δ*φ*_n_ and edge lengths Δ*l*_0_, Δ*l*_1_, …, Δ*l*_n_ (as shown in Figure 2), the first thing that need to be done is to trace clockwise around the outline of the polygon from some initial starting point to collect Cartesian (*x, y*) coordinates [35]. Once the coordinates of all the vertices are determined, they can be used to calculate edge lengths Δ*l_i_* and angular bend Δ*φ_i_*. The clockwise arc lengths *l* along the curve from starting point P_0_ up to the *i*th vertex P*_i_* is given as Equation (1)

(1) $$l=\overset{i}{\underset{k=1}{\sum }}\Delta l_{k}$$

Next step is to determine the angle bend Δφ*_i_* between adjacent polygon segments. Taking the adjacent polygon segments as a vector, the magnitude and orientation of the angle can be determined based on vector operation. For three adjacent point, P*_i_*_-1_(*x_i_*_-1_*,y_i_*_-1_), P*_i_* (*x_i_, y_i_*) and P*_i_*_+1_(*x_i_*_+1_*,y_i_*_+1_), the magnitude of Δφ_i_ can be calculated using the dot products of vector $\vec{P_{i-1}P_{i}}$ and $\vec{P_{i}P_{i+1}}$.

(2) $$|\Delta \varphi i|=\arccos\left(\frac{\vec{P_{i-1}P_{i}}•\vec{P_{i}P_{i+1}}}{|\vec{P_{i-1}P_{i}}||\vec{P_{i}P_{i+1}}|}\right)$$

where $\left|\Delta \varphi_{i}\right|$ is the absolute value of Δ*φ_i_*. The polarity of Δ*φ_i_* depends upon the sign of the cross products of the two vectors, which is given as the determinant of a matrix S*_i_* [36].

(3) $$S_{i}=|\begin{array}{ccc} x_{i-1} & y_{i-1} & 1\\ x_{i} & y_{i} & 1\\ x_{i+1} & y_{i+1} & 1 \end{array}|$$

If S*_i_* > 0, then $\vec{P_{i}P_{i+1}}$ is clockwise with respect to $\vec{P_{i-1}P_{i}}$, if S*_i_* < 0, it is counterclockwise. Once the sign of this cross product is determined, the value of Δ*φ_i_* can be obtained by applying the following rules.

(4) $$\mathrm{if}\;S_{i}\neq 0,\;\Delta \varphi_{i}=\left|\Delta \varphi_{i}\right|\times \frac{S_{i}}{\left|S_{i}\right|}$$

(5) $$\mathrm{if}\;S_{i}=0,\;\Delta \varphi_{i}=0$$

where $\left|S_{i}\right|$ is the absolute value of S*_i_*. It should be noted that a cross product of 0 means that vector $\vec{P_{i-1}P_{i}}$ and $\vec{P_{i}P_{i+1}}$. are collinear.

The cumulative angular function φ(*l*), also known as Z-R shape function, is the summation of all the angular bends from vertex P_0_ up to the vertex P*_i_* (corresponding to arc length *l*). This function is given by φ(*l*) = $\sum_{k=1}^{i}\Delta \varphi_{k}$. If the outline is traced clockwise and is not spiral, the value of φ(L_all_) equal to -2π, where L_all_ is the total length of the polygon. Throughout the above procedures described in Equations (1)–(5), the coordinates of points on the polygon are converted from their Cartesian (x,y) form to the φ(*l*) form of Z-R shape function [37]. Shape analysis can be conducted based on the result of Z-R shape function [31] (Figure 2).

### 2.2. Generation of Irregularly Shaped Polygon

**Figure 3:**
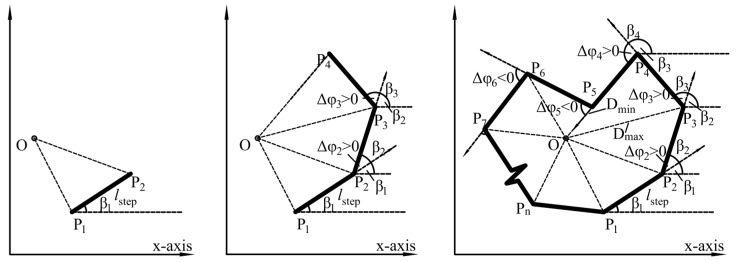
Procedures of generating randomly shaped polygon.

Inspired by the theory of Z-R shape function, the RAB algorithm is proposed in this section and coded in Python. This method is the inverse operation and modification of the theory of Z-R shape function. As shown in Figure 3, the algorithm can be described as follows.

1. Define a random point O as the center and a point P_1_ (*x*_1_, *y*_1_) as initial starting point of the polygon to be generated.
2. Determine the position of the sequential points to be generated. To determine the position of sequential points, a series of angular turns need to be conducted. The common approach to solve this problem is to treat the polygon segments as vectors. The counterclockwise angle that vector $\vec{P_{i}P_{i+1}}$ makes with the positive x-axis is β_i_. For i=1, β_i_ is a random number varying uniformly between 0 and 2π. For i >1, the value of this angel can be calculated by Equation (6)
  *β_i_* = *β_i_*_−1_ + Δ*φ_i_* (*i* >1) (6)
  where Δ*φ_i_* is the angle bend at point P*_i_*, which is treated as a uniformly distributed random variable. In the procedures of generating randomly shaped polygon, the orientation of the angle should be taken into account and are randomly assigned. For *i* >1, the coordinates of P*_i_* in a Cartesian coordinate system can be represented by Equation (7)
  (7) $$\{\begin{array}{c} x_{i}=x_{i-1}+l_{\mathrm{step}}\times \cos(\beta_{i})\\ y_{i}=y_{i-1}+l_{\mathrm{step}}\times \sin(\beta_{i}) \end{array}\;(i>1)$$
  where *l*_step_ is constant step length.
3. To ensure the points on the polygon are generated counterclockwise, the point P*_i_* should be at the left side of line OP*_i_*_-1_ or on the line (for example, point P_5_ in Figure 3). This can be validated using the cross product of vector $\vec{{\mathrm{OP}}_{i}}$ and $\vec{{\mathrm{OP}}_{i-1}}$. The calculation of the cross product is conducted through Equation (3). If this condition is satisfied, proceed with step 4; otherwise, repeat from step 2.
4. Calculate D_OP_*_i_* —the distance between O and the generated point P*_i_*(*x_i_, y_i_*). There are two parameters required to be inputted in advance in this part. They are the minimum distance D_min_ and the maximum distance D_max_ between the center O and the generated points. If the distance is within the specified range (D_min_ ≤ D_OP*i*_ ≤ D_max_) then the point is stored as a new vertex. Otherwise repeat from step 2.
5. If a point cannot be generated within specified times of trials, then remove the vertex stored in the previous step and repeat from step 2.
6. The summation of all the angular bends from the initial starting point and the point generated in the previous step is *φ* (*φ*=∑Δ*φ_i_*). In case of the generation of spiral polygon (a spiral polygon is a simple polygon whose boundary chain contains exactly one concave subchain [38]), φ should equal to 2π [39]. Consequently, repeat steps 2, 3, and 4 until the value of φ is larger than or equal to 2π.

If the value of *φ* satisfies the convergence criteria in step 6, then delete the last point and connect the stored vertexes counterclockwise. Thus, individual polygon with irregular shape is successfully generated. If necessary, the above steps are repeated until the specified amount of polygon are obtained. It should be noted that the distance between point P_n_ and P_1_ may not equal to *l*_step_. However, this situation does not have a significant influence on the polygon contour.

## 3. Analysis of the Generated Particle Shape

### 3.1. Shape Descriptors

Many attempts have been made to quantitatively analyze the geometrical characteristics of particles in the literature. Based on the dimensional scales, the morphological characteristics of these particles are expressed in terms of three major aspects: form (overall shape), roundness (angularity), and surface texture (roughness) [26,40,41].

Form, the property of the first level, represents spatial irregularities of particle shape in the large dimension. The common shape include circle, ellipse, rectangle, etc. Shape descriptors, such as elongation index (EI), aspect ratio (AR), and flatness index (FI) are used to quantify the basic shape characteristic of particle. The EI can be determined as follows Equation (8) [22]

**Figure 4:**
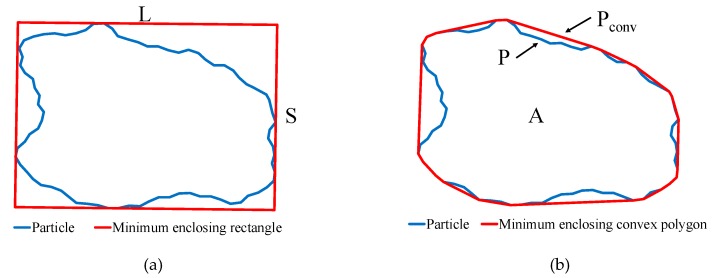
Schematic diagram of shape descriptors: EI, RI, and RE: (**a**) Diagram of the EI; (**b**) Diagram of the RI and RE.

**Figure 5:**
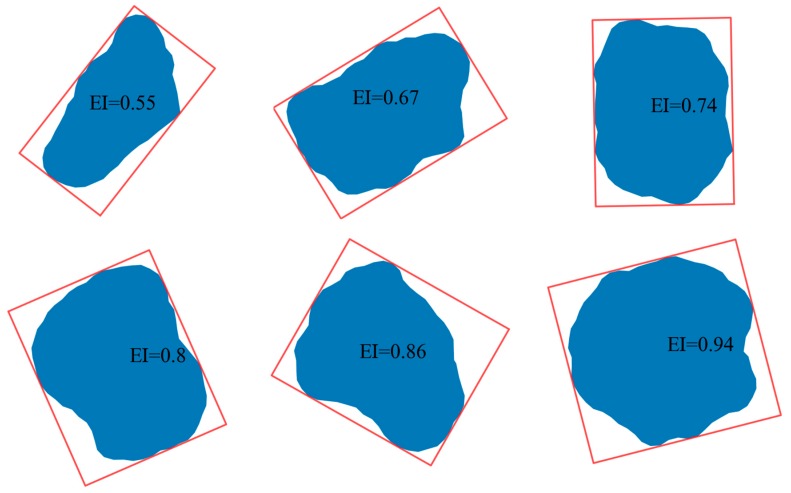
Particles with different elongation index.

EI = S / L(8)

where S and L equal to the width and length of the smallest rectangular box containing the particle, respectively (Figure 4a). The elongation index varies from 0 to 1, where the higher value of EI indicates a lower degree of elongation. For a circle, the EI equals to 1. Particles with different elongation index are shown in Figure 5.

**Figure 6:**
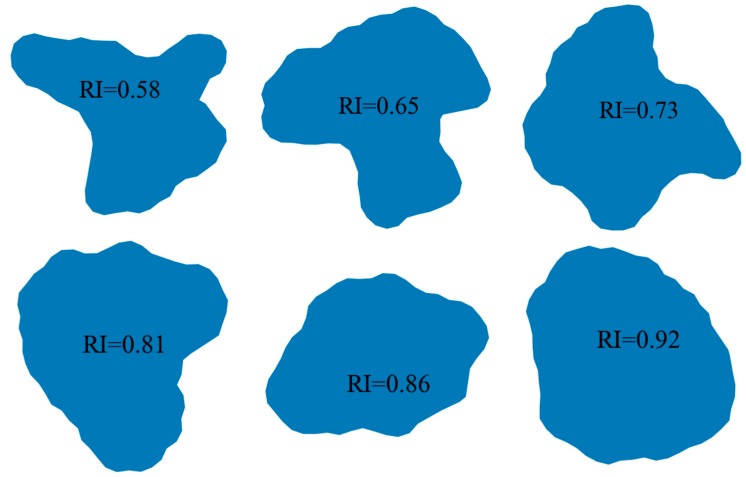
Particles with different roundness.

Roundness—the property of the second level—describes the variations of particle shape in the medium dimension, which reflects the average sharpness at corners. It is independent of the overall shape. Available shape descriptors including angularity index, roundness index (RI) and others are of this expression. The degree of roundness is defined and calculated via the following Equation (9) [42].

(9) $$\mathrm{RI}=\frac{4\pi A}{P^{2}}$$

where A is the projected area and P is the perimeter of the particle contour (see Figure 4b). Figure 6 displays six particles generated using the proposed algorithm. It is obvious that as RI increase from 0.58 to 0.92, high rounded particles can be obtained (Figure 6).

**Figure 7:**
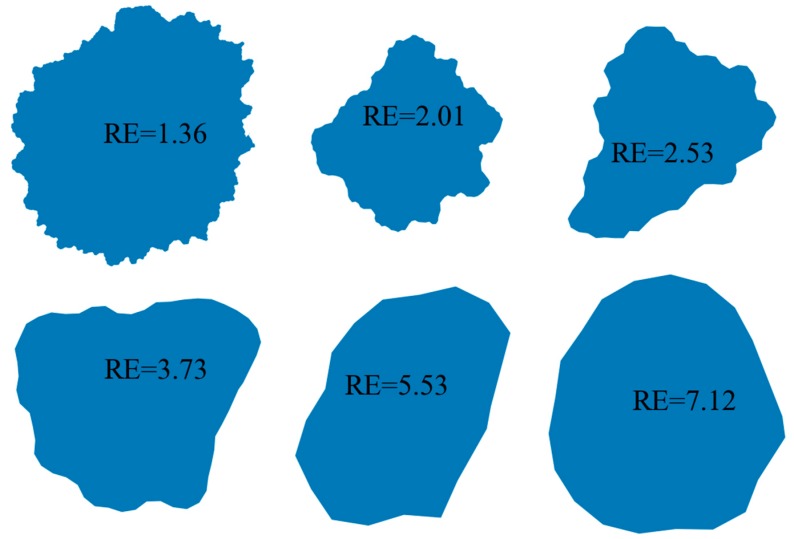
Particles with different regularity.

Surface texture, the property of the third level, refers to the surface features which are small-scale relative to the size of the object [41]. Roughness index and regularity index are generally used to describe the surface texture features. The regularity index (RE) is defined as Equation (10) [22]

(10) $$\mathrm{RE}=\log\left(\frac{P}{{P-P}_{\mathrm{conv}}}\right)$$

where P is the perimeter and P_conv_ is the convex perimeter of the particle (see Figure 4b). The value of regularity greater than 3 or 4 indicated the particle surface is very smooth. The example particles shown in Figure 7 illustrate this point.

From Figure 5, Figure 6 and Figure 7, it is obvious that three shape descriptors mentioned above—elongation index, roundness index and regularity index—have a good performance in obtaining the multiaspect characteristics of particle shape (form, roundness, and surface texture of a particle outline). So these three shape descriptors are chosen for the quantitative shape analysis in the following section.

### 3.2. Relationship between Input Parameters and Shape Descriptors

There are several parameters required to be inputted manually in RAB algorithm. They are the minimum distance D_min_ and the maximum distance D_max_ between the center O and the generated points, the range of angle bend Δφ*_i_* and the step length *l*_step_ (see Figure 3). In this paper, the minimum value of Δφ*_i_* is set to 0 and the step length is treated as a constant. To better control the shape of generated particle, based on the geometric relationship between these parameters, these parameters are summarized and classed into three representative parameters, which are R_dist_—the ratio between D_min_ and D_max_, R_l/d_—the ratio between *l*_step_ and D_max_ and the maximum value of angle bend Δφ_max_ (see Equations (11)–(13)).

R_dist_ = D_min_ / D_max_ (11)

R_l/d_ = *l*_step_ / D_max_ (12)

(13) $$\Delta \varphi_{\max}≧\mathrm{Max}(\left|\Delta \varphi_{1}\right|,\;\left|\Delta \varphi_{2}\right|,\ldots ,\;\left|\Delta \varphi_{n}\right|)$$

**Figure 8:**
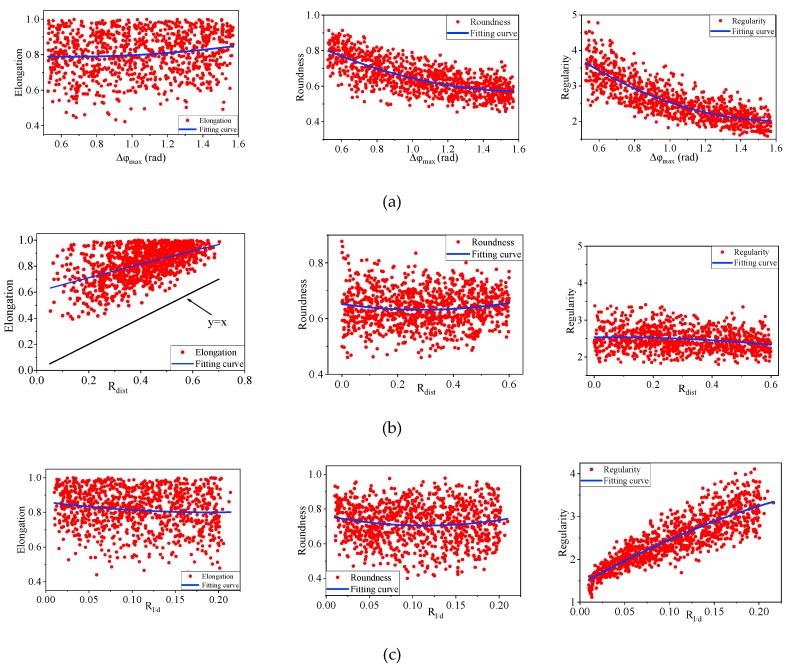
Influence of input parameters on shape descriptor: (**a**) Influence of Δφ_max_ on particle shape; (**b**) Influence of R_dist_ on particle shape; (**c**) Influence of R_l/d_ on particle shape.

To explore the influence of these three parameters on shape descriptors (EI, RI and RE), each of them is considered as a random variable, within a specified range. Based on the range of R_dist_, R_l/d_, Δφ_max_, which are provided in Table 1, 1000 irregularly shaped polygons are generated for each group. The values of shape descriptors are obtained using Equations (8)–(10), respectively. Figure 8 presents the effect of the three representative parameters on particle shape. The influence can be determined through the dispersion degree of the data points, which is obtained by range analysis. The greater dispersion degree indicates the input parameter has marginal control effect on corresponding shape descriptors. By comparison of the graphs in the same column, combined with the dispersion degree of data point, the effect of three representative parameters on shape descriptors are investigated through qualitative analysis. Besides, some fitting curves are almost horizontal in Figure 8, indicating that the control effect of corresponding parameters on a certain shape descriptors is rather limited or can be neglected.

Figure 8a illustrates the influence of parameters Δφ_max_ on the generated particle shapes. It is obvious that Δφ_max_ is well correlated with the particle roundness and regularity. Nevertheless, an increase of Δφ_max_ has limited effect on the particle elongation. However, with the increase of R_dist_, the variation of shape descriptors is different with the above situation (see Figure 8b). Based on the results of shape analysis, the EI of generated shape is almost always greater than that of corresponding R_dist_. Thus, in the process of generating random particles, R_dist_ can be used to control the minimum value of elongation, while R_dist_ has limited influence on RI and RE. Finally, the discrete degree of the data points shown in Figure 8c reflects the influence of R_l/d_. It can be observed that this parameter have negligible influences on elongation and roundness. Nevertheless, increase of R_l/d_ leads to a significant increase of particle regularity.

**Figure 9:**
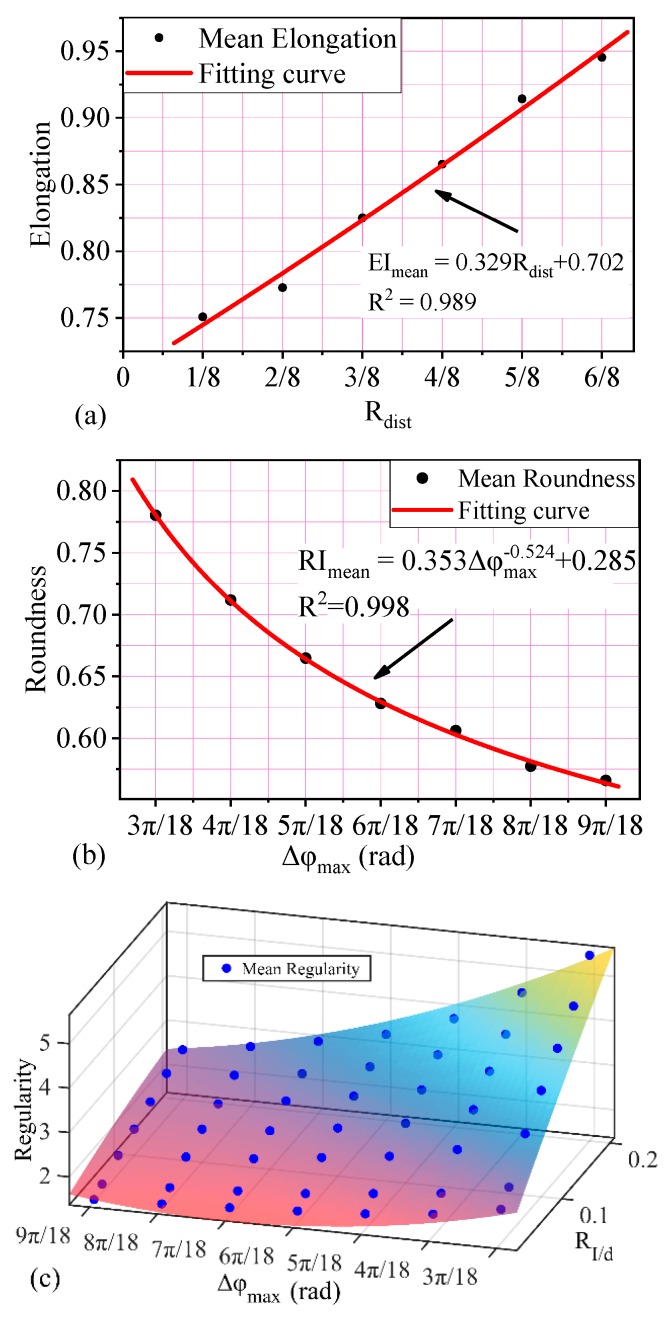
Relationship between input parameters and shape descriptor: (**a**) Relationship between input parameters and EI_mean_, (**b**) relationship between input parameters and RI_mean_, and (**c**) relationship between input parameters and RE_mean._

Based on the analysis above, the three shape descriptors are controlled by single or multiple input parameters with an obvious effect. The relationship between input parameter and shape descriptor is obtained by using the curve fitting method (Figure 9). The correlation coefficient R^2^ is adapted to evaluate fitting precision [43,44]. Among these shape descriptors, the minimum and mean value of elongation (EI_min_ and EI_mean_ ) are determined by R_dist_ (Figure 9a), which can be expressed as

EI_min_ ≧ R_dist_ (14)

EI_mean_ = 0.329R_dist_ + 0.702 (15)

Besides, as shown in Figure 9b, the Δφ_max_ has a statistically significant relationship with the mean value of roundness (RI_mean_). The value is calculated based on the following fitting formula.

RI_mean_ = 0.353Δφ_max_^−0.524^ + 0.285 (16)

Different from EI and RI, the mean value of regularity (RE_mean_) is controlled by both Δφ_max_ and R_l/d_ (Figure 9c). The predictive formula could be obtained by the curve fitting method with R^2^ =0.989 and expressed as follows

RE_mean_ = 3.43 + 23.99R_l/d_ − 3.578Δφ_max_ − 6.985 R^2^_l/d_ − 11.57 R_l/d_ · Δφ_max_ + 1.504Δφ^2^_max_ (17)

Thus, in the procedure of generating random particles, the shape of particles can be quantitatively controlled based on Equations (14)–(17).

## 4. Overlap Detection Algorithm

After a series of arbitrarily shaped polygons are introduced, the generated geometric models are import into the Particle Flow Code PFC^2D^ (Version 5.0., Itasca Consulting Group Inc.: Minneapolis, MN, USA) [33] to construct clump library. PFC^2D^ is a two-dimensional DEM software. The particles are treated as disks or clumps, and can both translate and rotate. The movement of particle obeys Newton’s laws of motion. Based on the mid-surface method, the clumps are created to represent realistic particle shapes [34] and appropriately packed in the model domain. To facilitate random and quick allocation of irregularly shaped clumps, an overlap detection algorithm is proposed in this section. This algorithm can be summarized as follows.

**Figure 10:**
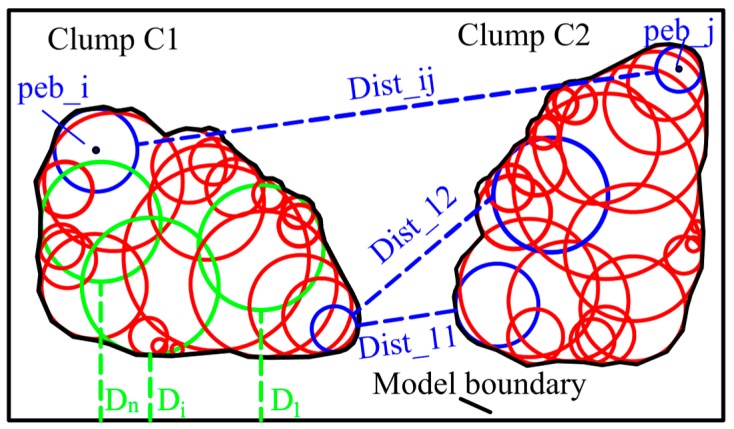
Schematic diagram of distance calculation method.

1. Construct a clump library based on the randomly generated polygons.
2. Select a clump from the library and specify the position randomly in the model domain. It is obvious that the shape of a clump is determined by the position and radii of the comprising pebbles. Based on the pebble information, the minimum distance between the clump and the boundary of model can be determined by calculating the distance (D_i_) between each pebble and the model boundary (see Figure 10).
3. Detect overlap between the clump and model boundary. If the minimum distance between them is greater than or equal to zero, it suggest that the clump does not intersect with the boundaries of model. If this condition is satisfied, store the clump information; otherwise, repeat from step 2.
4. Put next clump in the model domain and calculate the minimum distance between this clump and the packed clumps (or the model boundary) based on the position and radii of all the pebbles. If this clump do not overlap with the packed clumps or exceed the model boundary, store the clump and pebble information; otherwise, repeat this step.
5. Repeat step 4 until a given condition is satisfied (e.g., the number of all the clumps, the area of all the clumps).

The most important part of the algorithm is the calculation of the minimum distance between clumps or the distance between clump and the model boundary. This is an iterative procedure in which the minimum distance is determined by calculating the distance between pebbles in new selected clump and pebbles in the packed clumps (and the distance between pebbles in new selected clump and model boundary). As shown in Figure 10, the distance between clumps C1 and C2 is defined by Equation (18):

Dist(C1,C2) = Minimum(Dist_11, Dist_12, Dist_13…, Dist_ij)(18)

where Dist_ij is the distance between pebble peb_i in clump C1 and pebble peb_j in C2. In a similar way, the distance between clump and model boundary can be calculated. Thus, in the procedures of clump packing, the distance between each clumps in the model domain can be precisely controlled.

## 5. Application of Proposed Algorithm

To validate the feasibility of the proposed RAB and overlap detection algorithms in modeling of granular materials, two group of granular mixture samples for application examples are generated in the following sections.

### 5.1. Modeling of Granular Mixture Materials

**Figure 11:**
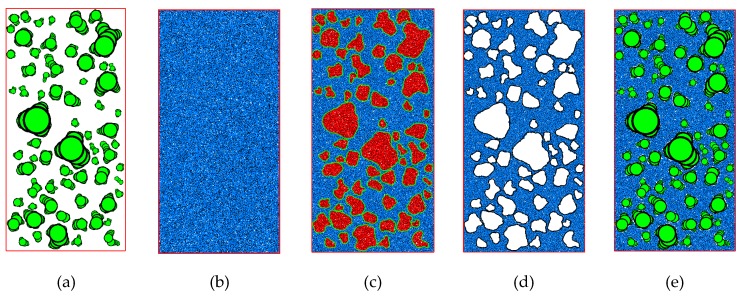
Procedures of generating granular mixtures: (**a**) Put coarse particles into the model domain, (**b**) generate fine particles, (**c**) overlap detection (the red regions), (**d**) delete fine particles in the red regions, and (**e**) generate granular mixtures.

Granular mixtures are inhomogeneous multiphase materials, which are consisted of coarse particles and fine particles [45]. In this numerical model, the coarse particles are simulated using clumps of overlap pebbles and fine particles by single disk. The procedures of modeling granular mixture are as follows (Figure 11).

1. Code the RAB algorithm in Python and input parameters R_dist_, R_l/d_, and Δφ_max_ to simulate the geometric model of individual coarse particle with irregular shape.
2. Import the generated geometric model into PFC^2D^ to construct clump library. The clumps are created based on mid-surface method. These clumps are used to represent realistic coarse particle.
3. Put coarse particles into the model domain based on the overlap detection algorithm which is proposed above (Figure 11a).
4. Generate sample that is composed of single disk to simulate fine particles in the same model domain (Figure 11b). The size of particle and the porosity of fine particles specimen should be in a given range.
5. Remove fine particles that overlap with coarse particles (Figure 11c,d). The generated granular mixture sample is shown in Figure 11e.

### 5.2. Granular Mixture with Predetermined Coarse Particle Shape Features

**Figure 12:**
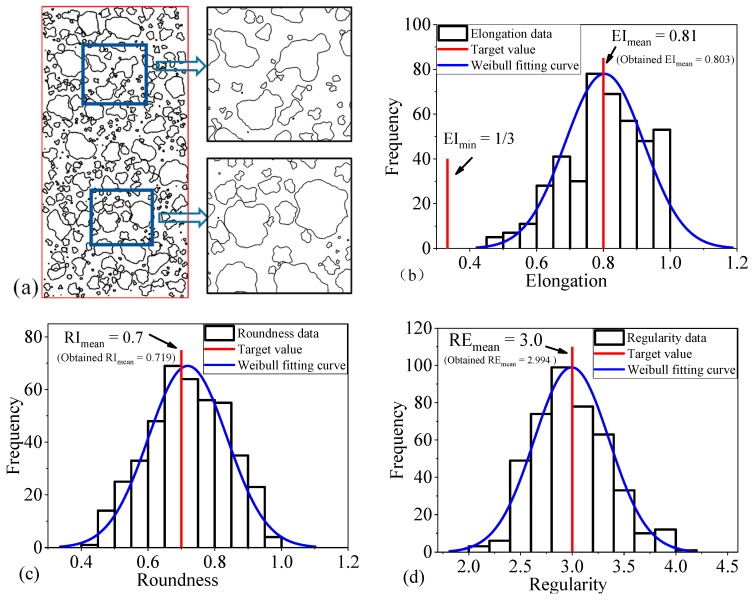
Granular mixture with predetermined coarse particle shape features: (**a**) Sample of generated granular mixture, (**b**) elongation distributions of coarse particles, (**c**) roundness distributions of coarse particles, and (**d**) regularity distributions of coarse particles.

The relationship between input parameters (R_dist_, R_l/d_, and Δφ_max_) and shape descriptors are discussed in Section 3.2, as well as the input parameters used to control particle shape features (such as the mean value of some shape descriptors). This section illustrates the capabilities of the proposed method in generating sample with predetermined shape feature. In this example, the mean and minimum elongation are equal to 0.81 and 1/3, respectively. The value of mean roundness (RI_mean_) and mean regularity (RE_mean_) are set to 0.7 and 3.0, respectively. Based on Equations (14)–(17), the three representative parameters (R_dist_, R_l/d_, and Δφ_max_) are back-calculated. The values are 1/3, 0.094, and 0.735, respectively. Besides, the equivalent diameters of the coarse particles varied uniformly from 0.01 to 1.0 cm and the coarse particles content is specified as 40%. It should be noted that because the clumps are used to represent realistic coarse particles shapes. Therefore, the area of all the coarse particles is obtained by calculating the area of clumps not that of randomly generated polygons. A granular mixture sample with predetermined shape feature and the derived distributions of the three shape descriptors (elongation, roundness, and regularity) are shown in Figure 12. It can be observed that the shape descriptors have a good match with the target value, which validates the feasibility of the RAB algorithm and three representative parameters in precisely controlling the generation of granular materials.

### 5.3. Granular Mixture with Different Coarse Particle Distances

**Figure 13:**
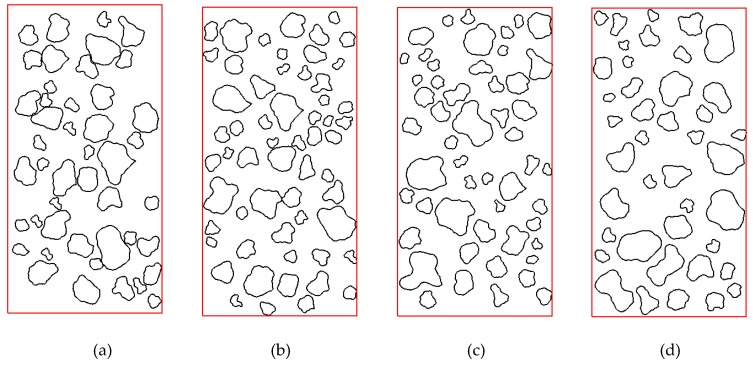
Granular mixture samples with different minimum coarse particle distances (cm): (**a**) d_min_ = 0.0 cm; (**b**) d_min_ = 0.05 cm; (**c**) d_min_ = 0.10 cm; (**d**) d_min_ = 0.20 cm.

**Figure 14:**
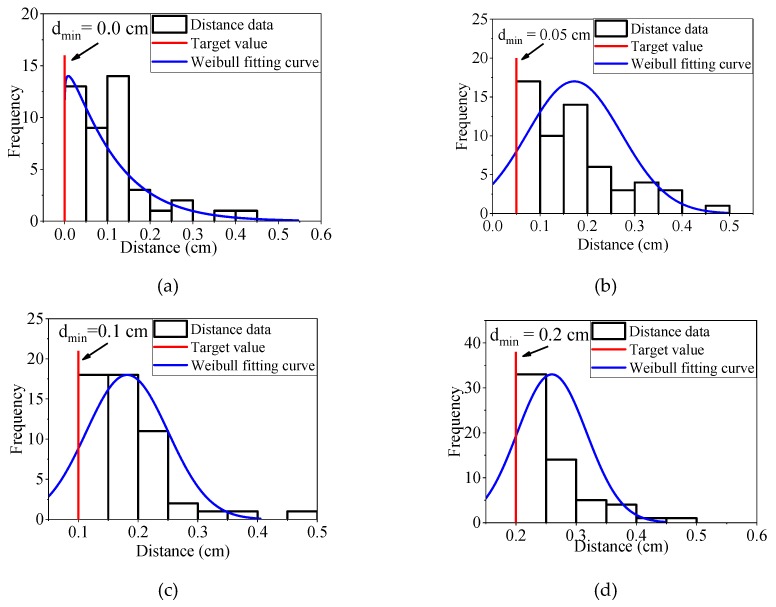
Frequency histograms of minimum distance between coarse particles: (**a**) d_min_ = 0.0 cm; (**b**) d_min_ = 0.05 cm; (**c**) d_min_ = 0.10 cm; (**d**) d_min_ = 0.20 cm.

The proposed overlap detection algorithm has an advantage in calculation of the minimum distance between coarse particles or the distance between coarse particle and the model boundary. To illustrate the capability of this algorithm, four granular mixture samples with different minimum coarse particle distances are generated (Figure 13). For these four samples, the minimum distance (d_min_) between coarse particles are 0, 0.05, 0.10, and 0.20 cm, respectively. The values of Δφ_max_, R_dist_, and R_l/d_ are set to π/4, 1/3, and 0.1, respectively. The value range of equivalent diameters are 0.3 to 1.5 cm. The content of the coarse particles is equal to 30%. The distributions of minimum distance between clumps are shown in Figure 14. All these results show that distance meet the requirements of the target value.

## 6. Conclusions

This paper presents a systematic method to generate arbitrary two-dimensional particle for the numerical simulation of granular materials. A random angular bend (RAB) algorithm is proposed and coded in Python to simulate the geometric model of individual particle with irregular shape. Three representative parameters (R_dist_, R_l/d_, and Δφ_max_) are used to quantitatively control the shape feature of generated polygons in terms of three major aspects (form, roundness, and surface texture). Besides, a novel overlap detection algorithm is developed to address difficulties associated with spatial allocation of irregularly shaped particles. Finally, two examples are further employed to validate the feasibility of the proposed algorithm for the numerical modeling of realistic granular materials. The main conclusions are summarized as follows.

1. The proposed RAB algorithm shows a good performance in the generation of randomly shaped polygons (especially for nonstar-like particle reconstruction based on image information).
2. Three representative parameters (R_dist_, R_l/d_, and Δφ_max_) in RAB algorithm could quantitatively control the shape feature of generated polygons by control three shape descriptors (elongation index, roundness index, and regularity). Besides, these three parameters have definitude physical meaning, which makes the RAB algorithm easier to understand.
3. The proposed overlap detection algorithm is able to allocate particle to the model domain easily by calculating the minimum distance between coarse particles or the distance between coarse particle and the model boundary.

It should be noted that the RAB algorithm is only suitable for the generation of two-dimensional particle. For real 3D particle modeling, this algorithm needs to be extended to a general 3D case in future works. The study provide a foundation for the construction of granular materials based on angular bend theory.

## Figures and Tables

**Table 1:** Ranges of input parameters.

Group	Number of Particles	Δφ_max_ (rad)	R_dist_	R_l/d_
Group 1	1000	[π/6, π/2]	1/3	1/10
Group 2	1000	π/3	[0,3/5]	1/10
Group 3	1000	π/3	1/3	[0.01, 0.2]

## List of symbols

*l*	Arc length from point P_0_ up to the vertex P*_i_*
*φ*	Cumulative angular bends
P*_i_*	The *i*th vertex on the polygon
Δ*l*_i_	Edge length
Δ*φ_i_*	Angular bend at vertex P*_i_*
*L_all_*	The total length of the polygon.
*β_i_*	The counterclockwise angle that vector $\vec{P_{i}P_{i+1}}$ makes with the positive *x*-axis
*l*_step_	Constant step length
EI	Elongation index
S	The width of the smallest rectangular box containing the particle
L	The length of the smallest rectangular box containing the particle
RI	Roundness index
A	The projected area of the particle
P	The perimeter of the particle
RE	Regularity index
P_conv_	The convex perimeter of the particle
D_OP*i*_	The distance between O and the generated point P*_i_*
D_min_	The minimum distance between the center O and the generated points
D_max_	The maximum distance between the center O and the generated points
R_dist_	The ratio between D_min_ and D_max_
R_l/d_	The ratio between *l*_steplength_ and D_max_
Δ*φ*_max_	The maximum value of angle bend
EI_min_	The minimum value of elongation
EI_mean_	The mean value of elongation
RI_mean_	The mean value of roundness
RE_mean_	The mean value of regularity
D_i_	The distance between pebble and the model boundary
Dist_ij	The distance between pebble peb_i in clump C1 and pebble peb_j in C2
d_min_	The minimum distance between coarse particles
